# Transcriptomic Analyses Reveals Molecular Regulation of Photosynthesis by *Epichloë* endophyte in *Achnatherum inebrians* under *Blumeria graminis* Infection

**DOI:** 10.3390/jof8111201

**Published:** 2022-11-14

**Authors:** Yue Zhu, Shibo Zhu, Fang Zhang, Zhenrui Zhao, Michael J. Christensen, Zhibiao Nan, Xingxu Zhang

**Affiliations:** 1 State Key Laboratory of Grassland Agro-ecosystems, Key Laboratory of Grassland Livestock Industry Innovation, Ministry of Agriculture and Rural Affairs, College of Pastoral Agriculture Science and Technology, Lanzhou University, Lanzhou 730020, China; 2 Jiayuguan Municipal Bureau of Agriculture and Rural Affairs, Jiayuguan 735100, China; 3 Retired Scientist from Grasslands Research Centre, Private Bag 11-008, Palmerston North 4442, New Zealand

**Keywords:** *Epichloë* endophyte, *Achnatherum inebrians*, *Blumeria graminis*, photosynthesis, transcriptomes

## Abstract

Photosynthesis is essential for the growth of all green plants, and the presence of an *Epichloë* endophyte enhances the photosynthesis of *Achnatherum inebrians* (drunken horse grass, DHG), including when it is under attack by fungal pathogens. However, few studies have examined the mechanism of the increased photosynthetic activity at the molecular level of *A. inebrians* when it is under pathogen stress. The present study investigated the effects of the presence of the *Epichloë* endophyte on the net photosynthetic rate, intercellular CO_2_ concentration, stomatal conductance, and transpiration rate of DHG plants under a *Blumeria graminis* infection condition, and we compared the transcriptomes using RNA sequencing. The results showed that the photosynthetic rate of *Epichloë* endophyte-infected (E+) plants was higher under the *B. graminis* infection condition, and also without this pathogen, when it was compared with *Epichloë* endophyte-free (E-) plants. The E+ plants uninfected with *B. graminis* had 15 up-regulated unigenes that are involved in photosynthesis which were compared to the E- plants that were uninfected with this pathogen. This suggests that the presence of an *Epichloë* endophyte up-regulates the genes that are involved in the process of photosynthesis.

## 1. Introduction

Plants living in complex environments interact closely with a wide range of microbes, including pathogens with different lifestyles and infection processes, and this, the plant’s health is challenged [1]. *Blumeria graminis* is a fungus of the Erysiphaceae, with a wide range of hosts, including 634 Poaceae species. The host grasses include important grain species such as wheat, barley, rye and oats [2]. *Blumeria graminis,* the cause of powdery mildew, is considered to be the sixth most important fungal phytopathogen in the world [3], infecting the leaves, stems, flowers and fruits of plants. This pathogen causes severe economic losses in food and pasture production [4]. *Blumeria graminis* is a strictly obligate biotrophic fungus [5,6], whose conidia, which are formed in fragile chains on plant surfaces, are spread by the wind. The germ tubes of germinating conidia form appressoria on the surface of the leaves from which infection pegs penetrate the wall of the epidermal cells and form haustoria that invaginate the plasmalemma within the penetrated cells. The haustoria enable the extraction of water and nutrients, without killing the penetrated epidermal cells. The export of the effector molecules to the host suppresses the immune response of the plant and accelerates the infection cycle [7], rapidly producing large numbers of conidia to efficiently complete multiple infections [7,8]. The infection process of *B. graminis* is ongoing and may lead to the extensive colonization of the host plants. This process not only absorbs a large amount of nutrients from the host, but it also seriously reduces the photosynthesis rate of the host plant [9,10], enhances respiration and transpiration, but significantly reduces the chlorophyll content, ultimately leading to a reduction in host plant’s carbon assimilation. Therefore, the growth and development of plants are hindered, and the yield is reduced. In severe cases, powdery mildew can lead to the death of the host plant [9,11,12].

*Epichloë* fungi form symbiotic, typically mutualistic relationships with nearly 30% of cool-season grasses across the globe [13,14,15]. Poaceae and *Epichloë* endophytes have evolved to form host-fungi-specific relationships that confer protection for the grass against various stresses [16,17,18], and in exchange, the plant provides the fungal endophyte with an ongoing supply of nutrients, a stable protected habitat, and transmission to the next generation through the seed of host grasses. An important characteristic of the relationship between the *Epichloë* endophytes and host grasses is that the growth of the two symbionts is fully synchronized [19]. *E. gansuensis* [20] or *E. inebrians* [21] is present in nearly 100% of *Achnatherum inebrians* plants (drunken horse grass, DHG), which is a perennial grass growing in natural grasslands in the alpine and sub-alpine regions in Northwestern China [20,22,23,24,25]. The association between DHG and *E. gansuensis* provides enhanced persistence, in part due to the production of alkaloids that cause livestock poisoning (ergonovine and ergine), and the deterrence of grazing [26,27,28]. It is the effect of these alkaloids that has given this grass the common name of drunken horse grass. An *Epichloë* endophyte can improve the tolerance of DHG to drought [29,30,31], salt [32], cold [33], heavy metal stress [34], pests [35] and pathogens [36,37,38].

Photosynthesis is the most fundamental metabolic activity in all green plants, and it plays an important role in the growth and development of plants. It is through photosynthesis that plants can produce and store nutrients. Photosynthesis is severely affected in all of its phases by different stresses. Since the mechanism of photosynthesis involves various components, including photosynthetic pigments and photosystems, the electron transport system, and the CO_2_ reduction pathways, damage at any level caused by a stress may reduce the overall photosynthetic capacity of a green plant [39,40,41]. Both abiotic [31,42] and biotic [37,43,44,45] stresses can influence the photosynthesis of plants. Under drought and flooding stress, the growth of the plants will be hindered, and the photosynthetic capacity of the plants will be reduced [31,42]. Under pathogenic stress, the photosynthesis of the plants is altered. Leaf pathogens reduce the photosynthetic parameters of the diseased leaves, including by reducing the green leaf area of the leaves [37,46,47]. Leaf rust pathogens, obligate biotrophic fungi, colonize the leaves, forming pustules, reducing the green leaf area of the plant and the chlorophyll content, thereby reducing the photosynthetic activity in the infected leaves [43]. The net photosynthetic rate of the light saturation is reduced by the reduced total photosynthesis [44]. The chlorophyll content and net photosynthetic rate of DHG were significantly reduced under the stress of ergot disease [45]. Under the infection of *B. graminis*, the chlorophyll content, the net photosynthetic rate, and the dry matter content of DHG were significantly reduced, but the intercellular carbon dioxide concentration was enhanced [37]. However, when an *Epichloë* endophyte is present in stressed plants, this stress is mitigated.

Xia et al. (2016) found that although powdery mildew inhibits the photosynthetic parameters of DHG, the presence of the *Epichloë* endophyte can reduce the damage caused by *B. graminis* [37]. Zhang et al. (2022) also found that the *Epichloë* endophyte significantly increased the chlorophyll content and decreased the intercellular carbon dioxide concentration of DHG under ergot disease stress conditions, thereby increasing the net photosynthetic rate of DHG under ergot stress conditions [45]. Researchers have found that the presence of an *Epichloë* endophyte enhances the resistance of ryegrass that is inoculated with the pathogens *Alternaria alternata, Ascochyta leptospora, Curvularia lunata and Fusarium avenaceum* [48]. Under drought stress conditions, the presence of an *Epichloë* endophyte increased the growth, biomass, and photosynthetic rate of DHG. At the transcriptional level, compared with DHG without an *Epichloë* endophyte, in DHG plants with an *Epichloë* endophyte, the participation of the genes involved in photosynthesis are mostly up-regulated, such as *PetH*, *Lhcb1* and *PsbQ*, and these genes are also up-regulated under drought conditions [31]. Ambrose and Belanger (2012) also noted that 13% of genes up-regulated in the presence of an *Epichloë* endophyte were involved in the photosynthesis process of red fescue (*Festuca rubra*) [49]. However, in recent years, the research on the mechanism of the stress that is conferred by pathogens of DHG has mainly focused on the physiological and biochemical aspects, and there is little information at the gene level of the photosynthetic response of DHG under pathogen stress conditions.

Therefore, this study mainly explored the changes of the genes involved in the photosynthetic process of DHG (*Epichloë* endophyte-infected, E+; *Epichloë* endophyte-free, E-) under *B. graminis* infection. To test our predictions, we measured the photosynthetic indicators in the E+ and E- DHG plants and identified specific photosynthesis-related genes that are associated with changes in the photosynthetic rates. The aim of this study was to determine the changes in photosynthetic indexes and the related genes that are involved in photosynthetic processes in *Epichloë*-mediated DHG plants that are infested with *B. graminis.*

## 2. Materials and Methods

### 2.1. Plant Material and Experimental Design

The seeds of *A. inebrians* plants host to the *E. gansuensis* endophyte were generated in 2011 from plants originating in a natural grassland with 100% *Epichloë* infection rate in the Sunan county of the Gansu province of China. Before sowing, the collected seeds were divided into two parts, and one part was treated with thiophanate-methyl fungicide to ensure that the seeds were free of a viable *Epichloë* endophyte; the other part was not treated. These two seed types were planted in an experimental field of the College of Pastoral Agriculture Science and Technology, Yuzhong campus of Lanzhou University (104°39′ E, 35°89′ N, Altitude 1653 m) in 2012 [36]. The seed samples harvested from plants of the treated and untreated plants were stained with aniline blue and microscopically examined to determine the *Epichloë* endophyte status and ensure that the seeds with 100% and 0% infections were used in this study.

From 14 October to 14 December 2021, a pot experiment was conducted in the greenhouse of the College of Pastoral Agriculture Science and Technology, Yuzhong campus of Lanzhou University. On 14 October, 3 plump E+ and E- seeds were sown into each of the 240 pots (120 pots E+ and 120 pots E-, diameter: 24 cm; height: 15 cm), which were filled with vermiculite (75 g) that had been sterilized in an oven at 190 °C for 2 h. The pots were randomly placed in the constant temperature greenhouse (temperature: 26 ± 2 °C, humidity: 42% ± 2%) and watered sufficiently until the surface of the vermiculite was moist. After the emergence of the second fully expanded leaf, Hoagland’s solution was applied every 7 days.

The inoculation of the DHG plants with the pathogen *B. graminis* took place when the inflorescences had emerged (from the above 240 pots, 200 pots with consistent and healthy growth plants were selected for the treatment). The inoculation process was carried out by smearing 5 mL of a suspension of *B. graminis* at a concentration of 2 × 10^6^ spores per mL on the 50 E+ and 50 E- plants (pathogen-inoculated plants, P+ plants). Using a sterile brush, the spore suspension of *B. graminis* that was obtained above, was applied evenly on the surface of each leaf of DHG from tip to base. In the control group, the leaves of the 50 E+ and E- DHG plants were smeared with 5 mL of sterilized water (non-inoculated plants, P- plants). After the inoculation, the pathogen-inoculated and non-inoculated plants were kept in two separate greenhouse compartments under the same growth conditions. The plants were monitored for four weeks after the pathogen inoculation.

### 2.2. Measurement of Photosynthetic Data

On the morning of 11 January (four weeks after the pathogen inoculation), the photosynthetic indexes (including the net photosynthetic rate, the intercellular carbon dioxide concentration, the transpiration rate and the stomatal conductance) of the plants were measured using a photosynthesis instrument (L1-6400, LI-COR Nebraska, Lincoln, NE, USA). Ten pots with consistent levels of *B. graminis* infection were selected from each treatment, and three leaves from the same growth position were selected from each pot, with the top of each leaf being measured three times [31,37].

### 2.3. RNA Extraction and Transcriptome Analysis

At four weeks, three fresh leaves from each of three pots of the E+ and E- DHG plants that had been inoculated with *B. graminis*, and also, three pots of E+ and E- DHG plants that had not been inoculated were collected and stored in liquid nitrogen at −80 °C for the subsequent RNA extraction and transcriptome sequencing. The total RNA, 3 μg per sample, was extracted using the TRIzol reagent (Invitrogen, Carlsbad, CA, USA). The RNA extraction quality and integrity were assessed by 1% agarose gel electrophoresis and an Agilent 2100 Bioanalyzer (Agilent Technologies, Santa Clara, CA, USA). The mRNA-Seq Library was constructed using Illumina^®^’s NEBNext^®^ UltraTM RNA Library Prep Kit. The RNA sequencing was performed by the Biomarker Technologies Company (Beijing, China) [38].

High-quality clean reads (=90.54 Gb) were obtained from the raw reads by removing sequences containing adapters, poly-N, and low-quality sequences. The obtained unigene sequences (in FASTQ format) were used to query specific functions in the following databases: NR (NCBI non-redundant protein sequences); Pfam (Protein family); KOG/COG/eggnog (Clusters of Orthologous Groups of proteins); Swiss-Prot (a manually annotated and reviewed protein sequence database); KEGG (Kyoto Encyclopedia of Genes and Genomes); GO (Gene Ontology). The FPKM (Fragments Per Kilobase Million mapped reads) was calculated to estimate the expression level of all of the genes in each sample. The differentially expressed genes (DEGs) (FDR (false discovery rate) < 0.05 and |Log2fold change| ≥ 2) were analyzed using DEseq2_EBseq. Gene ontology (GO), enrichment, and the functional annotation of DEGs were implemented using the GOseq R package. The DEGs were further subjected to a Kyoto Encyclopedia of Genes and Genomes (KEGG) pathway analysis to identify the main biochemical metabolic pathways and signal transduction pathways.

### 2.4. Meta-Analysis Data Collection

The papers in journals that had been published up to 10 March 2022 were collected from the China National Knowledge Infrastructure (CNKI) and Web of Science databases. The search keywords were “*Epichloë*/endophyte/pathogens/photosynthesis” and “Grass endophytes, pathogens and photosynthesis” [50]. After multiple screenings, 46 articles were finally screened for the meta-analysis of this study, including 11 English articles and 35 Chinese articles (the references are attached to the supplementary materials).

GetaData Graph Digitizer 2.22 (http://getdata-graph-digitizer.com/, first access: 10 October 2021) was used to obtain the mean, standard deviation (if the standard error could be calculated according to the following formula) and the number of repetitions (n) on the graph of the net photosynthetic rate in the above literature; the data in the table were obtained directly. If the standard deviation could not be obtained directly, it was estimated according to 10% of the mean.

Standard deviation=standard error × √n

where *n* represents the number of repetitions of each treatment.

A total of 109 sets of observations were obtained from the selected 46 publications, and the obtained data were analyzed according to the following categories:

1. The net photosynthetic rate under the plant treatment with the *Epichloë* endophyte was selected and grouped according to different grass species. E+ (*Epichloë*-infected) was the experimental group; E- (*Epichloë*-free) was the control group.

2. The net photosynthetic rate under the treatment of plant pathogenic fungi was selected and grouped into E+ and E- groups. Pi+ (pathogen–inoculated, pathogens include *Blumeria graminis* and ergot) was the experimental group, and Pi- (non-inoculated) was the control group.

MetaWin software (version 2.1) was used to perform a meta-analysis to calculate these effects using the log of the response ratio (lnR) using the following Equation according to another study [51].

$$lnR=In\left(\frac{\bar{X_{e}}}{\bar{X_{c}}}\right)=In\left(\bar{X_{e}}\right)-In\left(\bar{X_{c}}\right)$$

where $\bar{X_{e}}$ and $\bar{X_{c}}$ represent the mean of the experimental group and the control group, respectively.

The variance of the natural logarithm of the response ratio was approximated by the following equation:

$$lnR\left(v\right)=\frac{S_{e}^{2}}{n_{e}{\bar{X}}_{e}^{2}}+\frac{S_{c}^{2}}{n_{c}{\bar{X}}_{c}^{2}}$$

$n_{e}$ and $n_{c}$ indicate the repetitions of the experimental and control groups, respectively, and $S_{e}$ and $S_{c}$ indicate the standard deviations of the experimental and control groups, respectively [52]. The lnR mean of the effect of the *Epichloë*/pathogen infection on the net photosynthetic rate of host grasses were calculated using the combination of lnR and lnR(v), where the lnR for each observation was weighted by the inverse variance of the lnR. The 95% bootstrap confidence intervals (95% CI) for the mean lnR were calculated using 9999 iterations of bootstrapping, according to a previous study [52].

### 2.5. Statistical Analyses

The differences in the photosynthetic indexes of the plants under the *Epichloë* endophyte and *Blumeria graminis* infestation conditions were tested using a two-way analysis of variance (ANOVA) using the datarium package of R software. A statistically significant two-way interaction was followed up by simple main effect analyses. All of the values are means ± SE of the mean.

## 3. Results

### 3.1. Plant Growth and Biomass

The *Epichloë* endophytes and *Blumeria graminis* had significant effects (*p* < 0.05) on the plant height, dry weight and fresh weight values of *A. inebrians*. In addition, there was a significant interaction (*p* < 0.05) between *A. inebrians* and *B. graminis* regarding the plant’s height (Table S1). However, there was no significant interaction between *Epichloë* and *B. graminis* for the tiller number, dry weight and fresh weight values (Table S1). The presence of the *Epichloë* endophytes significantly (*p* < 0.05) increased the plant height (Figure S1a), dry weight (Figure S1c) and fresh weight (Figure S1d) values. However, the inoculation with *B. graminis* significantly (*p* < 0.05) reduced the dry weight (Figure S1c) and fresh weight (Figure S1d) values of *A. inebrians*. Comparing E+ and E-plants that were infected by *B. graminis*, the presence of the *Epichloë* endophytes significantly (*p* < 0.05) increased the plant height of *A. inebrians* (Figure S1a).

### 3.2. Photosynthesis Index

**Figure 1:**
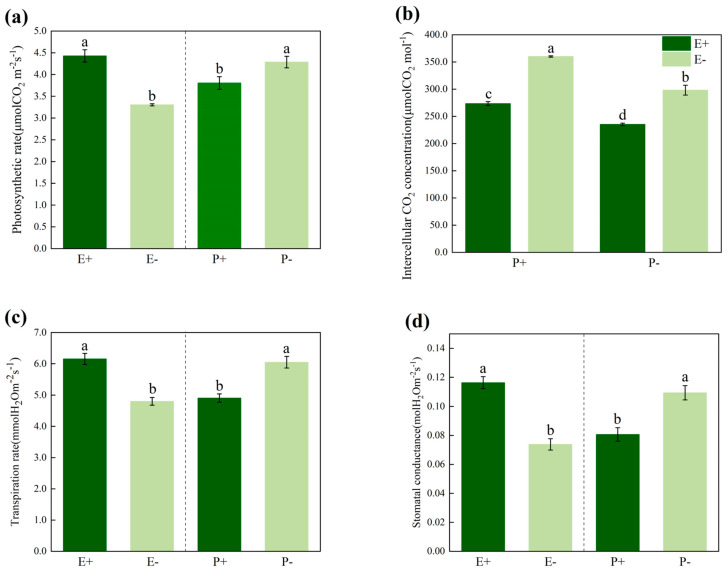
Net photosynthetic rate (**a**), intercellular CO_2_ concentration (**b**) transpiration rate (**c**) and stomatal conductance (**d**) of E+ (*Epichloë*-infected) and E- (*Epichloë*-free) *Achnatherum inebrians* plants under the P+ (pathogen-inoculated) and P- (non-inoculated). Values are mean ± standard error (SE), with bars indicating SE. Columns with non-matching letters indicate a significant difference at *p* < 0.05.

The *Epichloë* endophytes and *Blumeria graminis* had significant effects (*p* < 0.05) on net photosynthetic rate, intercellular CO_2_ concentration, transpiration rate and stomatal conductance of *A. inebrians*. In addition, there was significant (*p* < 0.05) interaction between the endophyte and *B. graminis* regarding the intercellular CO_2_ concentration (Table 1). However, there were no significant interactions between the endophyte and *B. graminis* for net photosynthetic rate, transpiration rate and stomatal conductance (Table 1). The presence of the *Epichloë* endophyte significantly (*p* < 0.05) enhanced the net photosynthetic rate (Figure 1a), transpiration rate (Figure 1c) and stomatal conductance (Figure 1d) of *A. inebrians*. However, the inoculation with *B. graminis* significantly (*p* < 0.05) reduced the net photosynthetic rate, transpiration rate and stomatal conductance of *A. inebrians*. Comparing the E+ and E- plants that were infected by *B. graminis*, the presence of the *Epichloë* endophyte significantly (*p* < 0.05) decreased the intercellular CO_2_ concentration of *A. inebrians* (Figure 1b).

### 3.3. Kegg Pathway Enrichment Analysis of the DEGs

**Figure 2:**
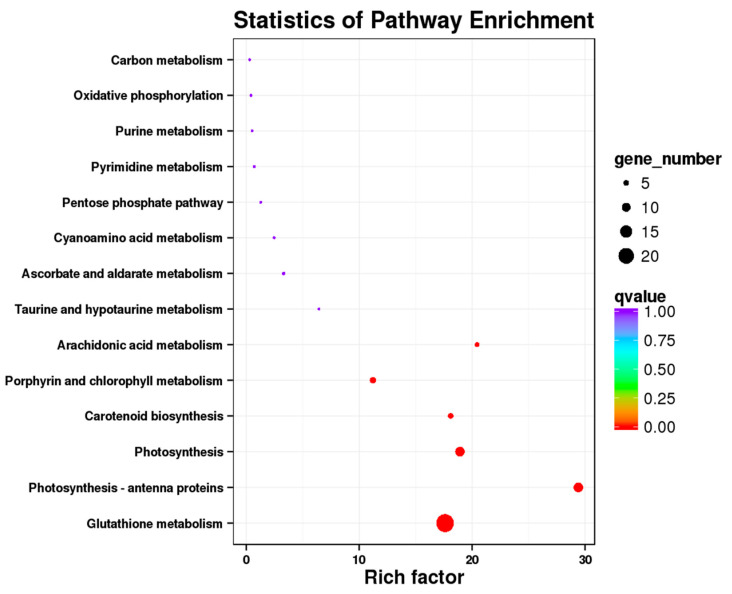
KEGG pathway enrichment of DEGs associated with *Achnatherum inebrians* under the E+ (*Epichloë*-infected), P+ (pathogen-inoculated) and both fungi (E+P+). Only the top 20 most strongly represented pathways are displayed in the diagram. The q-value ranges from 0 to 1, and a q-value that is closer to 0 indicates a greater enrichment.

A large number of differential genes were detected in this analysis, and these genes were mainly enriched in Glutathione metabolism (ko00480), Photosynthesis-antenna proteins (ko00196), Photosynthesis (ko00195), Prophyrin and chlorophyll metabolism (ko00860) (Figure 2). The photosynthetic genes were enriched in the photosynthetic pathway and in the antenna protein pathway. In this analysis, a total of 22 DEGs were detected in the Photosynthesis and Photosynthesis-antenna proteins.

### 3.4. DEGs of Regulatory Pathways Related to Photosynthesis

**Figure 3:**
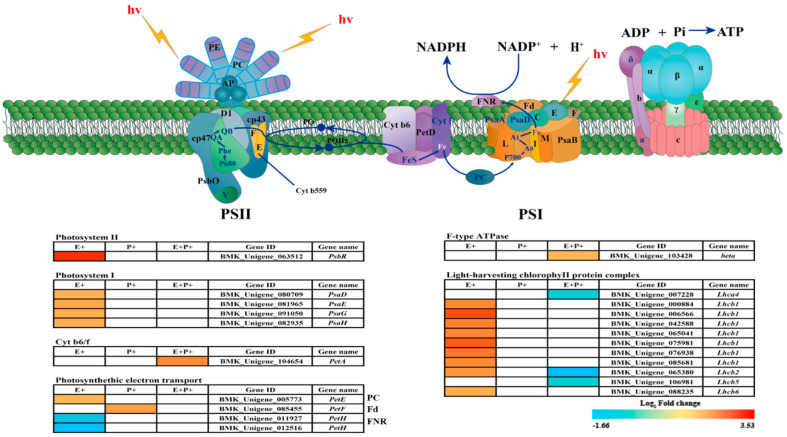
Photosynthesis-related DEGs in E+ (*Epichloë*-infected), P+ (pathogen-inoculated) and both fungi (E+P+) on *Achnatherum inebrians* plants. Red and blue colors represent the up-regulated and down-regulated proteins, respectively. Gene IDs and gene names are shown.

Single genes with fold changes in the gene expression that were greater than or equal to two and FDR values that were below 0.05 were defined as DEGs by a comparison with the E-P- treatment. Based on these stringent criteria, there were seventeen (fifteen up and two down) DEGs between E-P- and E+P-, five (two up and three down) DEGs between E-P- and E+P+, and between E-P- and E-P+, there was one (one up-regulated and zero down-regulated) DEG among the different overlapping differential genes under each treatment (Figure S2).Twenty-two DEGs were identified in the Photosynthesis and Photosynthesis-antenna proteins pathways, and these DEGs were found to be associated with the photosystem, membrane protein complex, photosynthetic electron transport chain, and antenna proteins (Figure 3 and Table S2). The presence of the *Epichloë* endophyte up-regulated fifteen unigenes and down-regulated two unigenes. Among these, the levels of four genes encoding the PSI protein complex, including *PsaD*, *PsaE*, *PsaG* and *PsaH*, as well as the PSII protein complex (*PsbR*) were improved in response to the presence of the *Epichloë* endophyte. Furthermore, nine unigenes encoding chlorophyII b binding protein *Lhcb1*, *Lhcb2* and *Lhcb6* of the light-harvesting complex were also observed to be up-regulated. In addition, one gene encoding the *PetE* protein involved in photosynthetic electron transport was down-regulated, whereas two unigenes encoding *PetH* were down-regulated. E- plants in the presence of *B. graminis* had one up-regulated gene; the unigene encoding the *PetF* protein that is involved in photosynthetic electron transport was up-regulated. E+ plants in the presence of *B. graminis* had two up-regulated genes, and three down-regulated genes. One gene encoding subunits of the cytochrome b6/f complex (*PetA*) and one unigene encoding the beta subunits of the F-type H^+^-transporting ATP synthase complex were up-regulated, while three genes encoding chlorophyll a/b binding protein *Lhca4*, *Lhca2* and *Lhca5* of the light-harvesting complex were also observed to be down-regulated (Figure 3).

### 3.5. Go Functional Enrichment Analysis of the DEGs

**Figure 4:**
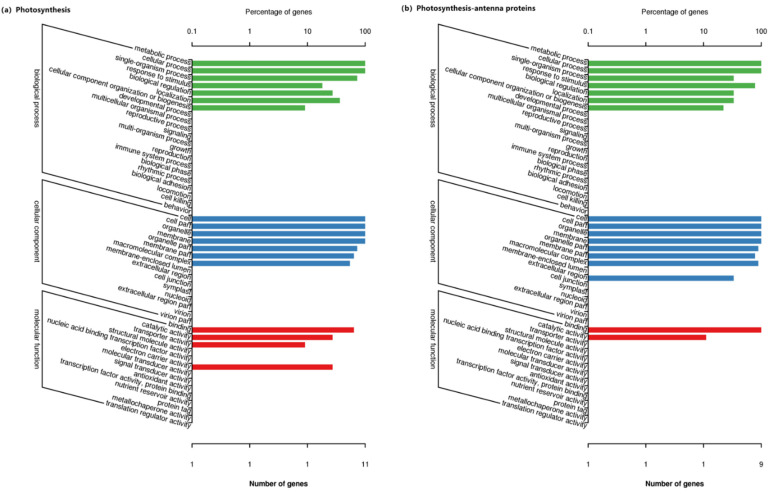
GO annotations of differentially expressed genes on the photosynthesis (**a**) and antenna protein (**b**) pathways of *Achnatherum inebrians* under each treatment.

There are 18 GO categories in the photosynthetic pathway (Figure 4a), and 17 GO categories in the antenna protein pathway (Figure 4b). There are a total of 22 DEGs in the photosynthetic and antenna protein pathways, and the detected genes were mainly divided into three categories: biological process, cellular component and molecular function. In the category of the biological processes, metabolic processes dominated, which was followed by cellular processes. In the category of the cellular components, cells, cell parts, organelles and membranes were predominant, which were followed by the organelle parts. In the molecular functional class, binding is the most important group, which is followed by catalytic activity (Figure 4).

### 3.6. Meta-Analysis

**Figure 5:**
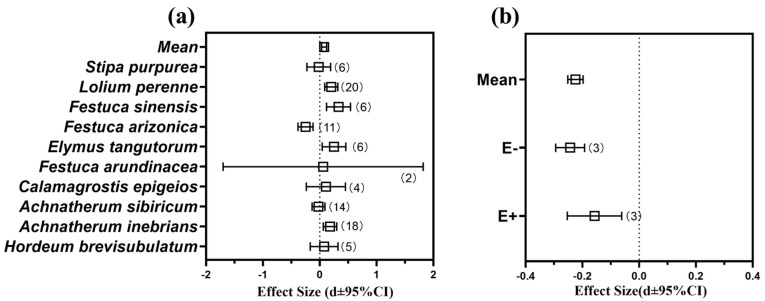
Effects on net photosynthetic efficiency (NPE) of grasses under the infestation of corresponding pathogens and *Epichloë* endophyte. (**a**) Effects of symbiotic *Epichloë* fungal endophyte on NPE of grasses (Relative effects of *Epichloë* symbiotic versus non-symbiotic plants in normal growth conditions) (**b**) Effects of inoculation with pathogens (include *Blumeria graminis* and ergot) on NPE of E+ (*Epichloë*-infected) and E- (*Epichloë*-free) grasses. Note: Error bars represent means ± bootstrap 95% CI. Note: The corresponding effect size and 95% CI are attached to the Supplementary Material Tables S3 and S4.

Based on the current research on the effect of the *Epichloë* endophyte on the net photosynthetic efficiency (NPE) of the host grass, the data of the host grass under normal growth conditions were selected for a meta-analysis, and there was an overall positive effect of the *Epichloë* endophyte on NPE of E+ plants which was observed when it was compared to E- plants (Qb = 153.3, *p* = 0.000, df = 9) (Figure 5a). The higher NPE values were observed in the *A. inebrians*, *Elymus tangutorum*, *Festuca arundinacea*, *F. sinensis*, *Calamagrostis epigeios*, *L. perenne* plants hosts of an *Epichloë* endophyte (Figure 5a and Table S3), and among them, the *F. sinensis* plants had the highest NPEs (effect size = −0.329, 95% CI = 0.119 to 0.540). However, the presence of an *Epichloë* endophyte was found to reduce the NPE of the host plants in the *A. sibiricum*, *F. arizonica* and *Stipa purpurea* plants (Figure 5a and Table S3).

There was an overall restrictive effect (main effect size = −0.225, 95% CI = from −0.251 to −0.198) of the phytopathogenic fungi on the NPE of the E- plants when they were compared to the E+ plants (Qb = 22.1, *p* = 0.000, df = 5) (Figure 5b). However, the NPE of the E+ plants were higher than they were in the E- plants under pathogenic fungal infestation condition (Figure 5b and Table S4).

## 4. Discussion

In the present study, transcriptomics was used to investigate the effect of the interaction between the *Epichloë* endophyte and the fungal pathogen *B. graminis* on the photosynthesis of DHG. The results showed that *B. graminis* weakened the photosynthesis of DHG, while the presence of *Epichloë* endophyte alleviated the damage of *B. graminis* on photosynthesis. Our study also indicated that the presence of an *Epichloë* endophyte upregulates some genes that are involved in the photosynthesis process.

### 4.1. Effects of Biotic and Abiotic Stresses on Photosynthesis

Our current study, which was conducted in the absence of an abiotic stress, we found that the *B. graminis* infection significantly reduced the photosynthetic rate, stomatal conductance and transpiration rate of the E+ and E- DHG plants, but the photosynthetic index of E+ plants was higher than it was in the E- plants (Figure 1). This is consistent with the findings of Xia et al. (2016) wherein the photosynthesis of DHG was inhibited both under the conditions of the artificial inoculation with *B. graminis* and the natural occurrence of powdery mildew [37]. Both biotic and abiotic stresses have been shown to reduce the photosynthetic rate by limiting the stomata or non-stomatal conductance [53,54], reducing plant growth and productivity [36]. For example, mild drought stress affects the photosynthesis, stomatal conductance, and water use efficiency in plant leaves [55,56,57,58]. Photosynthesis has a substantial contribution to the plant’s growth and development, and the chemical energy that is consumed in a wide range of metabolic processes in plants is mainly derived from photosynthesis. All green plants are capable of converting light energy into available chemical energy through photosynthesis [41]. However, the activation of the resistance responses in plants interacting with pathogens results in reduced crop yields and reduced photosynthetic parameters [46,47] when they are compared to uninfected plants. For example, leaves that are infected by *B. graminis* reduce the photosynthetic parameters of the plant, mainly by changing the stomatal conductance of the leaves and the concentration of carbon dioxide between the cells [59,60], which ultimately reduces the yield of the host plants [11,12].

### 4.2. Effects of Epichloë endophyte on Photosynthesis

As we expected, the results of the present study indicate that the *Epichloë* endophyte significantly enhanced the photosynthetic rate of DHG in both pathogen-infected and non-infected plants (Figure 1a). Another important finding of this study was that the *Epichloë* endophyte is an important indicator of altered photosynthesis in DHG (Table 1 and Figure 1). The largest number of DEGs related to photosynthesis were altered in the E+P- plants (Figure 3), and the E+ DHG plants that were not inoculated with *B. graminis* had higher growth rates than the non-inoculated E- DHG plants did (Table S1 and Figure S1). The presence of the *Epichloë* endophyte, growing intercellularly and synchronously with the surrounding tissue, from which it obtains all of its nutrients, should be a cost to the host plant [35,36,61]. However, the plant height, dry weight and fresh weight values were higher in the E+ DHG plants than they were in the E- plants in the heat-sterilized vermiculite, so the E+ DHG plants grew better than the E- plants did (Figure S1). The increased rate of photosynthesis due to the presence of the *Epichloë* endophyte not only compensated for the cost incurred by the presence of the systemic fungus, but it provided sufficient available nutrients to enhance the plant’s growth. Previous studies have demonstrated that plants that are symbiotic with the *Epichloë* endophyte have a higher biotic and abiotic tolerance [17,62,63,64]. The effects of biotic and abiotic stress on plant photosynthesis were mainly verified by measuring the photosynthesis indicators including the photosynthetic rate and the stomatal conductance. Meanwhile, the presence of an *Epichloë* endophyte improved the biomass and photosynthetic rate of DHG under different water treatments [29,31]. The current meta-analysis results indicate that the presence of an *Epichloë* endophyte can enhance the photosynthesis of the host grasses under pathogen stress conditions (Figure 5b). The results of meta-analysis also validate this point of view: the combination of plants with beneficial symbionts, including endophyte fungi, can alter the plant’s immune responses [65] and induce the plant defenses that enhance plant’s resistance to pathogens [66].

### 4.3. Presence of Epichloë endophyte Alters Photosynthetic Genes

Consistent with our hypothesis, the *Epichloë* endophyte can promote the photosynthesis of DHG by upregulating 17 genes encoding PSI, PSII, LHCI, LHCII, Cytochrome b6/f and ATP synthase (Figure 3). In this current study, 15 photosynthetic genes were up-regulated in E+P- DHG (Figure S2a). Unexpectedly, the expression level of the *PetH* gene encoding FNR in photosynthetic electron transport decreased in E+P- DHG (Figure 3), which blocked the electron transport process and inhibited photosynthesis. FNR is the terminal oxidase of the photosynthetic electron transport chain which receives electrons from Fd and H^+^ in the substrate and reduces NADP^+^ to NADPH. Similarly, the gene expression of the *PetH* protein involved in photoelectron transport was also down-regulated in *Dunaliella salina* under copper stress conditions [67].

Moreover, the abundance of light-harvesting chlorophyll of LHCI, LHCII protein in the E+ plants is higher than that in the E- plants, and the photosynthetic rate was higher in the E+ ones than it was in the E- plants (Figure 1a, Figure 3). Rozpądek et al. (2015) also noted that the presence of an *Epichloë* endophyte can improve the photosynthetic rate of *Dactylis glomerata*, and the abundance of the LHCI, LHCII protein in the E+ plants is higher than that in the E- plants [68]. The chlorophyll protein complex (LHC) plays an important role in the light harvesting of PSI and PSII, affecting the capture and transmission of light energy in the chloroplasts [69].

However, both chlorophyll complex protein I (*Lhca4*) and chlorophyll complex protein II (*Lhcb2, 5*) were down-regulated in E+P+ DHG (Figure 3). It may be that the presence of an *Epichloë* endophyte can only alleviate the pathogen stress to a certain extent, but this may not be a long-term effect. In this current study, the presence of an *Epichloë* endophyte also up-regulated the unigenes *PsbR*, *PsaD*, *PsaE*, *PsaG* and *PsaH* (Figure 3). *PsaD*, *PsaE*, *PsaG* and *PsaH* are subunits of the PSI-LHCI supercomplex, which are involved in light harvesting and electron transfer from plastocyanin (PC) to ferredoxin (Fd) [70,71].

The presence of the *Epichloë* endophyte is thought to shift the plant’s metabolism from defense to growth states, altering host transcript levels [33,68], enabling plants to efficiently use available resources for photosynthesis and carbon assimilation, and this enables the plants to obtain a higher biomass [31,37]. Under drought conditions, most of the selected unigenes involved in the photosynthetic process were up-regulated in *Epichloë* endophyte symbiotic DHG when they were compared with that of the non-symbiotic plants [31]. In addition, the presence of an *Epichloë* endophyte up- or down-regulates key genes in the biosynthesis and reaction pathways of jasmonic acid and salicylic acid under both biotic and abiotic stresses [30,38], and SA and JA can regulate complex immune responses to protect the plants from damage. In this analysis, 23 DEGs were enriched in the glutathione metabolism pathway (ko00480) (Figure 2). Glutathione has an important role in stress tolerance, and we speculate that the presence of an *Epichloë* endophyte promotes the production of defensive compounds for the plant’s protection.

In the current study, a total of 18 DEGs involved in photosynthesis were up-regulated under E+P+, E+P- and E-P+ conditions, of which 15 DEGs were in the E+P- plants. The transcriptome analysis showed that the presence of an *Epichloë* endophyte could significantly upregulate the genes related to photosynthesis.

## Figures and Tables

**Table 1:** Two-way ANOVA for the effects of *Blumeria graminis* (P) and *Epichloë* endophyte (E) on the photosynthetic rate, intercellular CO_2_ concentration, stomatal conductance and transpiration rate of *Achnatherum inebrians*.

Treatments	df	Photosynthetic Rate		Intercellular CO_2_ Concentration		Transpiration Rate		Stomatal Conductance	
		F	*p*	F	*p*	F	*p*	F	*p*
P	1	106.787	0.000	98.877	0.000	46.759	0.000	40.290	0.000
E	1	211.323	0.000	219.716	0.000	65.380	0.000	88.861	0.000
P × E	1	3.628	0.061	5.547	0.021	3.235	0.077	0.181	0.672

## Data Availability

All data supporting the findings of this study are available within the paper and within its supplementary materials published online. The RNA-seq used in this study have been deposited in the Squence Read Achieve (SRA) of the NCBI database under the accession number PRJNA748183.

## Supplementary Materials

The following supporting information can be downloaded at: https://www.mdpi.com/article/10.3390/jof8111201/s1, Table S1: Two-way ANOVA for the effects of Blumeria graminis (P) and Epichloë endophyte (E) on plant height, tiller number, dry weight fresh and weight of Achnatherum inebrians; Figure S1: Effects of Epichloë-infected (E+) and Epichloë-free (E-) on plant height (a), tiller number (b), dry weight (c) and fresh weight (d) under the pathogen-inoculated (P+) and non-inoculated (P-). Figure S2: Number of up-regulated (a) and down-regulated (b) plant genes associated with defense responses given the presence of Epichloë-infected (E+), the pathogen Blumeria graminis (P+) and both fungi (E+P+) on Achnatherum inebrians plants. E-: Epichloë-free, P-: non-inoculated; Table S2: Selected unigenes associated with processes of photosynthesis and photosynthesis-antenna proteins identified in the RNA-seq analysis in the present study; Table S3: Effect size of net photosynthetic efficiency (NPE) of different grasses under Epichloë endophyte infestation and Table S4: Effect size of net photosynthetic efficiency (NPE) of Epichloë-infected and Epichloë-free grasses under pathogenic fungi inoculation. References [72,73,74,75,76,77,78,79,80,81,82,83,84,85,86,87,88,89,90,91,92,93,94,95,96,97,98,99,100,101,102,103,104,105,106,107,108,109,110,111,112] are cited in Supplementary Materials.
