# Supplementary material for: Transcriptomic Analyses Reveals Molecular Regulation of Photosynthesis by Epichloë endophyte in Achnatherum inebrians under Blumeria graminis Infection
Source: J Fungi (Basel). 2022 Nov 14;8(11):1201. doi: 10.3390/jof8111201 (PMC9695909; doi:10.3390/jof8111201)
Supplement: Supplementary file 1 [file jof-08-01201-s001.zip › jof-1930875-supplementary.pdf]

Supplementary Materials

1. Plant Growth and Biomass

Table S1. Two-way ANOVA for the effects of *Blumeria graminis* (P) and *Epichloë* endophyte (E) on plant height, tiller number, dry weight, and fresh weight of *Achnatherum inebrians*.

**Treatments**	df	Plant heights		Tiller number		Dry weight		Fresh weight	
		F	*P*	F	*P*	F	*P*	F	*P*
P	1	11.984	**0.009**	4.052	0.079	18.391	**0.000**	61.607	**0.000**
E	1	39.271	**0.000**	0.122	0.736	12.660	**0.007**	18.035	**0.003**
P*E	1	6.417	**0.035**	0.092	0.770	3.235	0.232	0.087	0.643

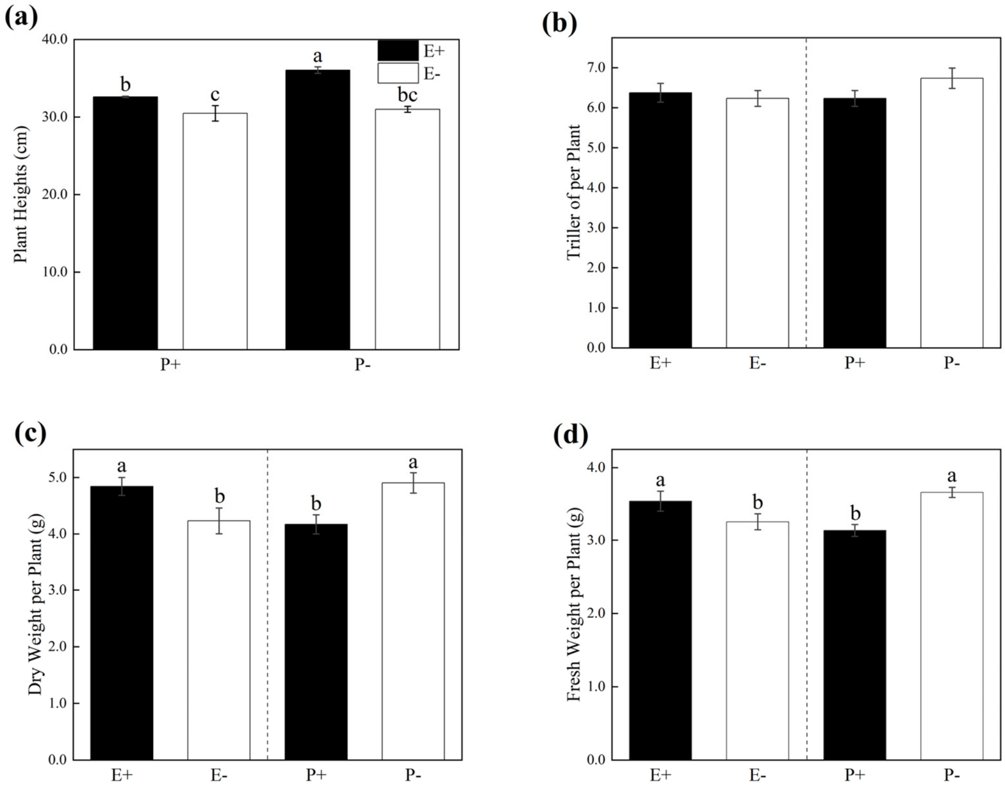

Figure S1: Effects of *Epichloë* endophyte-infected (E+) and *Epichloë* endophyte-free (E-) on plant height (a), tiller number (b), dry weight (c) and fresh weight (d) under the pathogen-inoculated (P+) and non-inoculated (P-). Values are mean±standard error (SE), with bars indicating SE. Columns with non-matching letters indicate a significant difference at $p<0.05$.

2. Differentially expressed gene (DEG) analysis

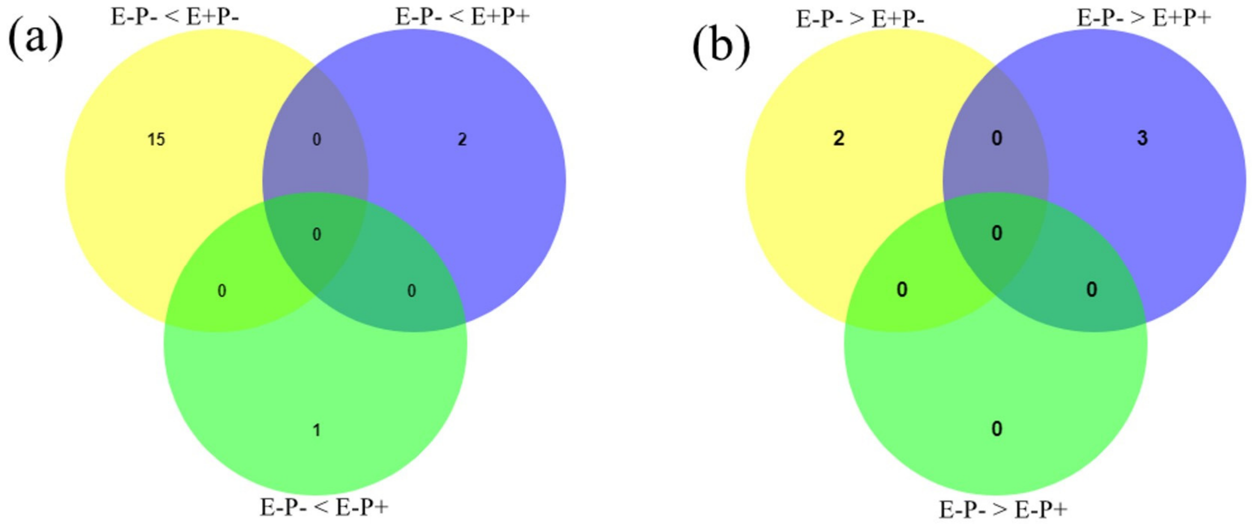

Figure S2: Number of up-regulated (a) and down-regulated (b) plant genes associated with defense responses given the presence of *Epichloë*-infected (E+), the pathogen *Blumeria graminis* (P+) and both fungi (E+P+) on *Achnatherum inebrians* plants. E-: *Epichloë*-free, P-: non-inoculated.

Table S2: Selected unigenes associated with processes of photosynthesis and photosynthesis-antenna proteins identified in the RNA-seq analysis in the present study.

ID	Primers (5'-3')	
	Forward	Reverse
BMK_Unigene_091050	TCCTCCTCCCCTCCACCACTAA	GCGCGCTCTTAGCGATTGGCCG
BMK_Unigene_005773	CCACCACCACCACCACCACCAC	CGCCGCCGCCAGACCGGGGCCC
BMK_Unigene_042588	GGCCTCACCGAACTTGACGCCG	GGGGGGAGAGGCGGAGCAGGCG
BMK_Unigene_082935	GTACGATTACTGATATCGACCA	TGAGACGCTTGGGTGAGGTTCG
BMK_Unigene_076938	AGGTAGCTCGGGGGCTCACCGG	ACACGGGACACCTCCAGTCTTA
BMK_Unigene_007228	ATACTGTCTCATCTCTTATTTT	TGGGGCACGGGTTGCACGGTAG
BMK_Unigene_075981	ACGGCCTCGCCGAACTTGACGC	GTGATGTGTCCCTCTCCGAACT
BMK_Unigene_085455	TCATGACAACAGTTTAAATTAG	CGATGATGGCCTCAGCGATTAG
BMK_Unigene_011927	GATCAAAACCCCTCTCCCCCCC	CTACAGAACATGTCACTTTCTG
BMK_Unigene_085681	AAGGAGATCAAGAACGGCCGCC	GTGATTGAAGCCCAGATCCGTG
BMK_Unigene_106981	GGTGAAGGAGATCAAGAACGGC	GCTCCACCCATGTTATAAAGAA
BMK_Unigene_012516	GGCAGTAATAGTTGGTCAGACT	CTGTTTTGTTCCACACTGCCAG
BMK_Unigene_065380	GGTGTCCCACCCGTAGTCACCG	GGCTGGTGTTCAAGTCCCCGCG
BMK_Unigene_081965	GAGAGTGAAATATGGAGAGTCT	ATGTGGATAACCTCCACTGTCG
BMK_Unigene_103428	GCCGGTAGCAAAGGTGGGTTAT	TTAGTATTTAGTTAGGTATTTG
BMK_Unigene_104654	TTTATCTGATTAGAGCGGAAAG	TTCTCTATCTAGAAGTGGAATA
BMK_Unigene_006566	GGAACTCAAGCCTCACTCTCGG	ACTGCGTGCCTTGCATTGCAAC
BMK_Unigene_088235	TTTGCTCTGAAATTTCCGGATA	CTTGAGCACGGCCGCGGAGGCG
BMK_Unigene_065041	CTCGAGAACCTCGCTGACCACA	CATTCTCCTGCGAGTACTCTCA
BMK_Unigene_000884	GCAAAAGATCAGTTTTTTCCAC	GTGGACGAGGCTGGGGTTGCCT
BMK_Unigene_080709	GTTACAGAATAAGTGTTGATCG	ACGTGGACGTCCCCCAAAGAGC
BMK_Unigene_063512	GCTGGACCCAAAGTATTCTGTT	TCACTCTTCCTCTAGTTGTCAG

Table S3: Effect size of net photosynthetic efficiency (NPE) of different grasses under *Epichloë* endophyte infestation.

Name	Effect size	95% confidence intervals (95% CI)
Mean	0.0760	0.0319 to 0.1200
Stipa purpurea	-0.0183	-0.2267 to 0.1901
Lolium perenne	0.2018	0.0866 to 0.317
Festuca sinensis	0.3293	0.1186 to 0.5399
Festuca arizonica	-0.25	-0.3824 to -0.1177
Elymus tangutorum	0.25	0.0429 to 0.4570
Festuca arundinacea	0.0589	-1.7008 to 1.8186
Calamagrostis epigeios	0.107	-0.2372 to 0.4512
Achnatherum sibiricum	-0.0229	-0.1359 to 0.09
Achnatherum inebrians	0.1801	0.0614 to 0.2989
Hordeum brevisubulatum	0.0761	-0.1676 to 0.3197

Table S4: Effect size of net photosynthetic efficiency (NPE) of *Epichloë*-infected (E+) and *Epichloë*-free (E-) grasses under pathogenic fungi inoculation.

Treatment	Effect size	95% confidence intervals (95% CI)
Mean	-0.2246	-0.2514 to -0.1978
E-	-0.2434	-0.2942 to -0.1926
E+	-0.1575	-0.2534 to -0.0616

Supplementary for Material and Methods

Meta-analysis

Reference:

[1] Bao, G. S.Effect of the hemiparasitic plant *Pedicularis kansuensis* on growth and photosynthetic properties of *Stipa purpurea-Epichloë* symbiosis. Lanzhou: Lanzhou University, 2015.

[2] Bao, G. S., Song, M. L., Wang, Y. Q., Li, C. J. Effects of parasitic *Artemisia annua* from Gansu on the photosynthetic characteristics of endophytic fungal symbiont in grasses. Acta Microbiol Sinica. 2020, 60, 294-305.

[3] Bao, G.S. Study of *Epichloë* endophyte-grass symbionts on photosynthetic characteristics of hemiparasitic plant. Chinese Qinghai Journal of Animal and Veterinary Sciences. 2020, 50, 1-7.

[4] Cao, Y. Effect of interaction between jasmonic acid and endophytic fungi on wild barely under salt stress. Lanzhou:Lanzhou University, 2018.

[5] Chen, T. X., Johnson, R., Chen, S. H., and Li, C. J. Infection by the fungal endophyte *Epichloë bromicola* enhances the tolerance of wild barley (*Hordeum brevisubulatum*) to salt and alkali stresses. Plant Soil 2018, 428, 353-370.

[6] Cui, X. L., Xia, C. Effect of exogenous abscisic acid on seedling establishment of *Epichloë gansuensis-Achnatherum inebrians* symbiont. Acta Prataculture Sinica. 2020, 29, 70-80.

[7] Deng, J. Mechanisms of the effects of arbuscular mycorrhizal fungus and grass endophyte on left spot of perennial ryegrass. Lanzhou:Lanzhou University, 2021.

[8] Deng, J., Li, F., Duan, T. Y. Effects of AM fungus and grass endophyte on the infection of *Lolium perenne* by the pathogen *Biopolaris sorokinianum* in a greenhouse. Acta Prataculture Sinica. 2019, 28, 103-113.

[9] Du, Y. J. Stress resistance research of endophyte in wild *Festuca L* and their association. Beijing:Beijing Forestry University, 2010.

[10] Du, Y. J., Sun, X. B., Han, L. B. Effect of *Neotyphodium starrii* infected on photosynthetic and orphological characteristics of tall fescue under high

temperature. Journal of Central South University of Forestry and Technology. 2010, 30, 41-47.

[11] Du, Y. J., Wang, Q., Han, L. B. Endophytic fungus *Neotyphodium typhinum* infection effects on photosynthetic characteristics of tall fescue. Ecol. Environ. Sci. 2009, 18, 45-47.

[12] Fang, A. G. Effect of *Neotyphodium* endophyte and AM fungi on growth of *Hordeum brevisubulatum* under salt and phosphorus stress conditions. Lanzhou:Lanzhou University, 2013.

[13] Guo, Y. E. Effect of phouphorus, *Claroideoglomus etunicatum* and grass endophte on perennial ryegrass left spot disease caused by *Bipolaris zeae*. Lanzhou:Lanzhou University, 2018.

[14] Guo, Y. E., Gao, P., Li, F. Effects of AM fungi and grass endophytes on perennial ryegrass *Bipolaris sorokiniana* leaf spot disease under limited soil nutrients. Eur. J. Plant Path. 2019, 154, 659-671.

[15] Guo, Y. E., Li, F., Wang, X. Y., Wang, Z. G., Duan, T. Y. Effects of phosphorus, AM fungi and grass endophytes on leaf spot of perennial ryegrass. Cao Xue. 2018, 4, 17-26, 72.

[16] Guo, Y. E., Li, Y.D., Gao, P., Wang, Z. G., Duan, T. Y. Effects of *Claroideoglomus etunicatum* and grass endophyte on the growth of *Lolium perenne* under different phosphorus levels. Acta Prataculture Sinica. 2018, 26, 1458-1466.

[17] Han, R., Li, X., Ren, A. Z., Gao, Y. B. Physiological ecological effect of endophyte infection on *Achnatherum sibiricum* under drought stress. Acta. Ecol. Sinica. 2011, 31, 2115-2123.

[18] Jia, T. Effect of endophytic species, host genotype and water/nutrient supply on the performance of grass-endophyte symbionts. Tianjing:Nankai Universitg, 2014.

[19] Jia, T., Ren, A. Z., Gao, Y. B. Host genotype overrides endophyte infection effects on growth, physiology, and nutrient content of a native grass, *Achnatherum sibiricum*. Plant Ecol. 2014, 215, 875-887.

[20] Jia, T., Ren, A. Z., Wang, S., Gao, Y. B. Effect of Endophytic fungi on growth and photosynthetic characteristics of *Achnatherum sibiricum*. Acta. Ecol. Sinica. 2011, 31, 4811-4817.

[21] Li, C., Li, X., Ren, A. Z., Gao, Y. B. Effect of endophyte infection on Zn resistance of *Achnatherum sibiricum*. Acta Scientiarum Naturalium Universititaatis Nankaiensis. 2013, 46, 29-35.

[22] Li, C., Ren, A. Z., Gao, Y. B. Effect of endophyte intection on Zn resistance of tall fescue. Acta. Ecol. Sinica. 2010, 30, 1684-1690.

[23] Li, F., Deng, J., Nzabanita, C. Growth and physiological responses of perennial ryegrass to an AMF and an *Epichloë* endophyte under different soil water contents. Symbiosis 2019, 79, 151-161.

[24] Li, F., Li, Y. Z., Duan, T. Y. Effects of interactions between a grass endophyte and an arbuscular mycorrhizal fungus on perennial ryegrass growth. Acta Pratculturae Sinica. 2017, 26, 132-140.

[25] Li, M. M. Effect of *Epichloë* endophyte on drought resistance of different ecotypes *Festuca sinensis*. Lanzhou:Lanzhou University, 2019.

[26] Li, N. N. Effect of interaction of endophyte and *Blumeria graminis* on the *Achnatherum inebrians* in different water conditions. Lanzhou:Lanzhou University, 2018.

[27] Li, X., Zhou, Y., Mace, W., Qin, J., Liu, H., Chen, W., Ren, A. Z., Gao, Y. B. Endophyte species influence the biomass production of the native grass *Achnatherum sibiricum* (L.) Keng under high nitrogen availability. Ecol. Evol. 2016, 6, 8595-8606.

[28] Liang, Y., Gao, Y. B., Chen, S. P., Ren, A. Z. Effects of endophyte infection on photosynthesis, transpiration and water use efficiency of *Lolium perenen* L. under drought stress. Acta Phytoecologica Sinica. 2001, 5, 537-543.

[29] Lin, F., Li, C., Zhang, X. Bao, X. Y., Ren, A. Z., Gao, Y. B. Effect of endophytic fungi on photosynthetic characteristics of three *Achnatherum sibiricum* populations in Inner Mongolia Steppe. Bull. Botanical. Res. 2009, 29, 61-68.

[30] Liu, B. H. Biological properties of endophtes/*Calamagrostis* symbiota. Naning:Naning Agricultural University, 2012.
[31] Liu, L. Response of *Epichloë gansuensis-Achnatherum inebrians* symbiont on stresses of cold and powdery mildew disease treated by SA and ABA. Lanzhou:Lanzhou University, 2016.
[32] Ma, M. Z. Disease resistance of perennial ryegrass (*Lolium perenne*)-*Epichloë festucae var. lolii* endophyte symbiont and their resistance mechanism to *Bipolaris sorokiniana*. Lanzhou:Lanzhou University, 2015.
[33] Ma, M. Z., Nan, Z. B. Effect of fungal endophytes against rust disease of perennial ryegrass (*Lolium perenne*) on growth and physiological indices. Acta Pratculturae Sinica. 2011, 20, 150-156.
[34] Morse, L. J., Day, T. A., Faeth, S. H. Effect of *Neotyphodium* endophyte infection on growth and leaf gas exchange of *Arizona fescue* under contrasting water availability regimes. Environ. Exp. Bot. 2002, 48, 257-268.
[35] Qin, J. H., Lu, Y., Li, Z., Zhou, Y., Ren, A. Z., Gao, Y. B. Effects of methyl jasmonate treatments and endophyte infection on growth of *Achnatherum sibiricum*. Chinese. J. Appl. Ecol. 2015, 26, 1145-1152.
[36] Rozpądek, P., Wężowicz, K., Nosek, M., Ważny, R., Tokarz, K., Lembicz, M., Miszalski, Z., Turnau, K. The fungal endophyte *Epichloë typhina* improves photosynthesis efficiency of its host orchard grass (*Dactylis glomerata*). Planta 2015, 242, 1025-35.
[37] Shi, Z. B., Zhou, Y., Li, X., Ren, A. Z., Gao, Y. B. Physioecological effects of endophyte infection on the host grass with elevated CO_2. Acta. Ecol. Sinica. 2013, 33, 6135-6141.
[38] Wang, J. F., Tian, P., Christensen, M. J., Nan, Z. B. Effect of *Epichloë gansuensis* endophyte on the activity of enzymes of nitrogen metabolism, nitrogen use efficiency and photosynthetic ability of *Achnatherum inebrians* under various NaCl concentrations. Plant Soil 2019, 435, 57-68.

[39] Wang, J. L., Gao, Y. B., Ren, A. Z., Wang, W., Zhao, N. X. Effects of endophyte infection on photosynthesis, transpiration and biomass of *Lolium perenne* L. at different nitrogen levels. Chinese Bull. Bot. 2004, 5, 539-546.
[40] Xia, C. Responses of *Epichloë gansuensis-Achnatherum inebrians* symbiont to drought stress. Lanzhou:Lanzhou University, 2018.
[41] Xia, C., Christensen, M. J., Zhang, X. X., Nan, Z. B. Effect of *Epichloë gansuensis* endophyte and transgenerational effects on the water use efficiency, nutrient and biomass accumulation of *Achnatherum inebrians* under soil water deficit. Plant Soil 2018, 424, 555-571.
[42] Xia, C., Li, N. N., Zhang, X. X., Feng, Y., Christensen, M. J., Nan, Z. B. An *Epichloë* endophyte improves photosynthetic ability and dry matter production of its host *Achnatherum inebrians* infected by *Blumeria graminis* under various soil water conditions. Fungal Ecol. 2016, 22, 26-34.
[43] Yan, Z. C., Li, Y. D., Cheng, W. J., Gao, P., Guo, Y., Duan, T. Y. Effects of AM fungi and grass endophyte on the growth of ryegrass under different salt concentrations. Grassland. Turf. 2018, 38, 63-70.
[44] Zhang, H. J. The effect of pathogenic ergot bacteria on the *Epichloë* endophyte symbionts of *Achnatherum sibiricum*. Lanzhou:Lanzhou University, 2021.
[45] Zhang, H. J., Li, X., White, J.F. *Epichloë* endophyte improves ergot disease resistance of host (*Achnatherum inebrians*) by regulating leaf senescence and photosynthetic capacity. Plant Growth Regul. 2022, 41, 808-817.
[46] Zhang, J. F. Effect of fungal endophyte on intraspecific competition of *Elymus nutans* under water stress. Lanzhou:Lanzhou University, 2013.
